# Analysis of Gastrointestinal Responses Revealed Both Shared and Specific Targets of Zinc Oxide and Carbadox in Weaned Pigs

**DOI:** 10.3390/antibiotics9080463

**Published:** 2020-07-30

**Authors:** Yuan-Tai Hung, Qiong Hu, Richard J. Faris, Juanjuan Guo, Pedro E. Urriola, Gerald C. Shurson, Chi Chen, Milena Saqui-Salces

**Affiliations:** 1 Department of Animal Science, University of Minnesota, St. Paul, MN 55108, USA; hungx121@umn.edu (Y.-T.H.); urrio001@umn.edu (P.E.U.); shurs001@umn.edu (G.C.S.); 2 Cargill Animal Nutrition, Elk River, MN 55330, USA; Qiong_Hu@cargill.com (Q.H.); Richard_Faris@cargill.com (R.J.F.); 3 Department of Food Science and Nutrition, University of Minnesota, St. Paul, MN 55108, USA; gjj0823@qztc.edu.cn (J.G.); chichen@umn.edu (C.C.)

**Keywords:** animal production, gastrointestinal tract, gut metabolites, cytokines, zinc oxide, carbadox

## Abstract

Antibiotics and pharmacological zinc supplementation were commonly used as growth promoters for several decades in the swine industry before being limited because of public health and environmental concerns. Further, the physiological and metabolic responses associated with their growth promotion effects are unclear. To characterize these responses induced by pharmacological zinc supplementation (2500 mg/kg) and carbadox (55 mg/kg), 192 post-weaning pigs were fed basal and test diets for 43 days. Compared with basal, pharmacological zinc and carbadox independently improved growth performance. Pharmacological zinc increased gastric mucosa thickness compared with basal zinc, while carbadox increased intestinal villus:crypt ratio compared with non-carbadox. Pharmacological zinc and carbadox independently reduced interleukin (IL)-1β concentration compared with basal zinc and non-carbadox. Pharmacological zinc increased IL-1RA:IL-1 ratio by 42% compared with basal zinc, while carbadox tended to increase the IL-10 and IL10:IL-12 ratio compared with non-carbadox. Carbadox increased fecal concentrations of histidine and lysine compared with non-carbadox. The independent effect of pharmacological zinc and carbadox on morphology and nutrient metabolism, and their shared effect on immunity may contribute to the additive effect on growth promotion. These results further confirmed the concept that growth promotion is multifactorial intervention. Therefore, elucidating growth-promoting effects and searching for alternatives should include wide-spectrum evaluation.

## 1. Introduction

Antibiotic growth promoters (AGP) and pharmacological doses of zinc (Zn, 1000–3000 mg/kg diet) from zinc oxide (ZnO) were the most common approaches to prevent the adverse effects of weaning in piglets. However, the non-therapeutic use of antibiotics as AGPs and pharmacological doses of Zn in weaned pigs is of concern because they may cause the selection of multi-drug resistant bacteria [1], and enterobacteria have been shown to develop resistant genes [2]. In addition, the excretion of Zn in manure can contaminate groundwater and soil [3,4]. Therefore, the European Union banned all in-feed AGPs in animal production in 2006 and will only allow the inclusion of dietary Zn concentrations of 150 mg/kg for supplementation to meet the piglets’ nutritional requirement by 2022. The United States (US) implemented the Veterinary Feed Directive in 2017, which prohibited the use of medically important antibiotics as growth promoters in animal diets [5]. Preventing post weaning diarrhea (PWD) and maintaining health and optimal growth performance of weaned pigs are priorities for pork producers. With the restriction of using AGPs and pharmacological doses of Zn in diets, various alternatives, such as probiotics, prebiotics, organic acids, enzymes, phytogenic compounds, and trace minerals, have been the subject of evaluation as growth promoters [6]. A recent review summarizing results from more than 2000 published trials showed limited improvements in weight gain of pigs fed diets using these alternative growth promoters [7]. Although the effects of AGPs and pharmacological doses of Zn on growth performance have been widely reported [8,9,10,11,12], the mechanisms involved in these effects have not been fully identified [13].

Several mechanisms of action of growth-promoting responses of Zn and AGPs have been proposed. Pharmacological Zn supplementation increased plasma ghrelin levels, altered gut microbiota, and maintained the intestinal mucosal barrier and immune system [14,15,16]. These responses have been suggested to contribute to growth promotion. Moreover, growth-promoting effects of AGPs may be attributed to antimicrobial properties, modification of gut microbiota, and a direct or indirect influence on inhibition of inflammatory responses [13,17]. However, in the industry these treatments are commonly used in combination, not separately. Whether pharmacological Zn supplementation and AGPs used in combination share common targets and mechanisms to exert their growth-promoting effects remains unclear. Animal growth is strongly related to nutrient digestion, utilization, and metabolism. Immunomodulation can also influence host growth response via its metabolic costs. Considering the significance of the gastrointestinal (GI) tract in these functions, our objective was to identify early morphological, immune and metabolic responses in the GI tract when feeding a pharmacological dose of Zn or carbadox, alone or in combination, in the diets of weaned pigs.

## 2. Results

### 2.1. Effects of Dietary Zn and Carbadox on Growth Performance

**Figure 1:**
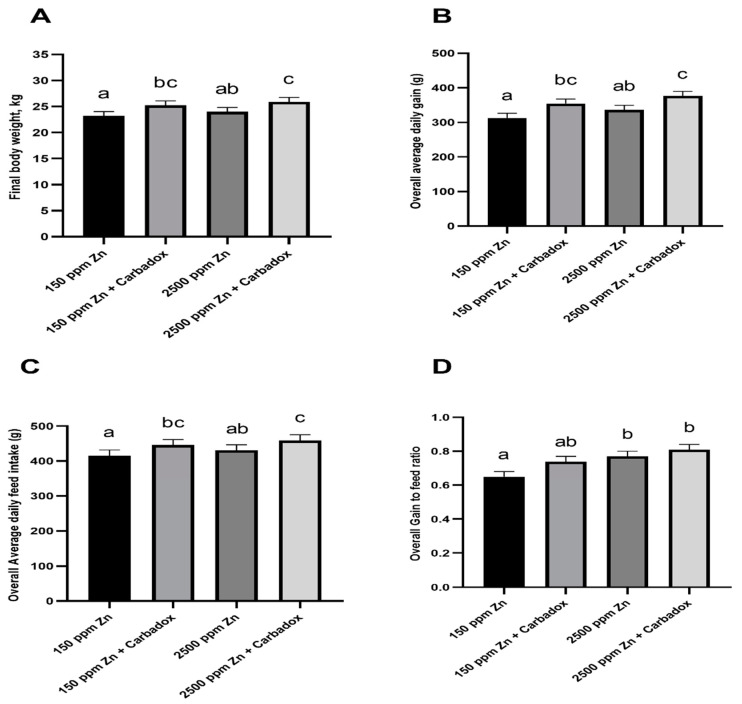
Final body weight (**A**), overall average daily gain (**B**), feed intake (**C**), and gain to feed ratio (**D**) of weaned pigs fed diets containing a pharmacological dose of Zn (2500 ppm) and carbadox. Different letters indicate significant differences (*p <* 0.05). Bars represent least squares means ± standard error of the mean.

The diets were formulated to fulfil the swine nutritional requirement (basal Zn, 150 mg/kg diet or with pharmacological Zn dose, 2500 mg/kg diet). Both diets were fed with or without carbadox (55 mg/kg diet). For overall growth performance responses, no interaction between Zn concentration and carbadox was observed (Figure 1). The time effect was significant (*p <* 0.05) for body weight (BW), average daily gain (ADG), average daily feed intake (ADFI), and gain to feed ratio (G:F), but there were no interactions of Zn × carbadox × time (Table S1). 

After the study period, pharmacological Zn supplementation numerically increased the BW of pigs by 3% compared with basal Zn, while carbadox supplementation increased *(p <* 0.01) the BW of pigs by 8% compared with non-carbadox treatments. Pigs fed diets containing the pharmacological dose of Zn plus carbadox were 12% heavier (*p <* 0.05) than pigs fed basal Zn without carbadox (control) (Figure 1A). For the 43-day feeding period, we observed that pharmacological Zn supplementation and carbadox treatments increased ADG by 7% (*p <* 0.01) and 13% (*p <* 0.01), respectively, compared with basal Zn and non-carbadox treatments (Figure 1B). Moreover, the combination of pharmacological Zn supplementation with carbadox resulted in an increase of ADG by 20% (*p <* 0.01) compared with pigs fed the control diet. These results indicated that pharmacological Zn dose and carbadox have an additive effect on growth rate.

Theoretically, ADFI is directly related to ADG responses in weaned pigs [18]. The overall results showed that pharmacological Zn supplementation tended to increase ADFI by 3% (*p =* 0.06) compared with the basal Zn, whereas carbadox addition to the diet increased ADFI by 7% (*p <* 0.01) compared with no carbadox added (Figure 1C). Additionally, the combination of pharmacological Zn supplementation and carbadox increased ADFI by 10% compared with feeding the control diet. For overall gain efficiency, pharmacological Zn supplementation and carbadox treatments improved G:F by 14% (*p <* 0.01) and 9% (*p =* 0.02), respectively, compared with basal Zn and non-carbadox treatments (Figure 1D). Furthermore, the combination of pharmacological Zn supplementation with carbadox improved G:F by 25% compared with pigs fed the control diet (*p <* 0.01). These results suggested a potentiating effect of pharmacological Zn dose and carbadox on gain efficiency.

### 2.2. Effects of Dietary Zn and Carbadox on Gastric Acid Production and GI Morphology

**Figure 2:**
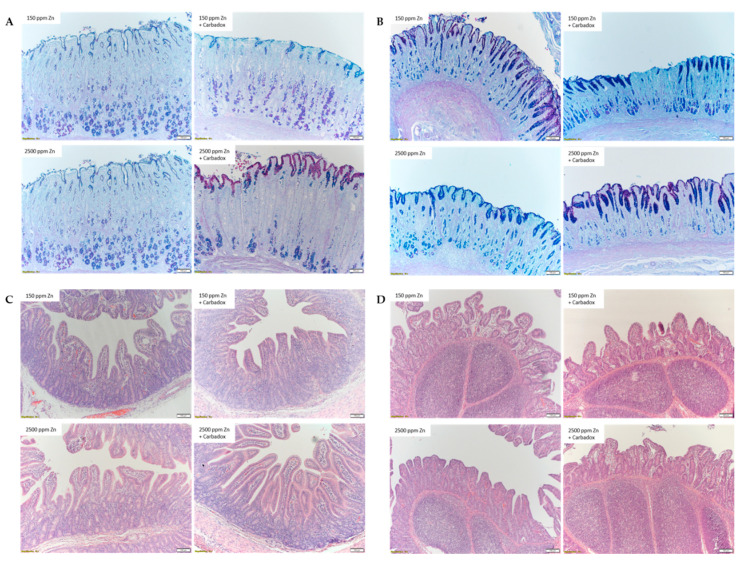
Representative images of gastric corpus (**A**) and antrum (**B**) stained with periodic acid–Schiff/Alcian blue and jejunum (**C**) and ileum (**D**) stained with hematoxylin and eosin (H&E). Scale bar: 100 µm.

Gastric and intestinal morphology were evaluated (Figure 2 and Table 1). There were no interactions between Zn concentration and carbadox on gastric morphology and gastric acid production (Table 1). Gastric morphology (Figure 2A,B) analysis showed that pigs fed pharmacological dose of Zn had a 14% and 11% greater (*p <* 0.01) mucosal height of the corpus and antrum, respectively, compared with those fed basal dose of Zn. In contrast, the addition of carbadox did not affect gastric mucosa. Both pharmacological Zn supplementation and carbadox treatments had no effects on the acidity of gastric contents, suggesting that the increase in the mucosa of the corpus was not related to changes in acid secretion.

The villus width and villus height of the jejunum and ileum of pigs were not affected by pharmacological Zn supplementation and carbadox treatments. However, interactions were observed for crypt depth of the jejunum and ileum, with pigs fed the carbadox diet having shallower crypts when in combination with the basal dose of Zn, but not with the pharmacological dose of Zn. More importantly, pigs fed diets containing carbadox had an 11% increase (*p <* 0.01) in villus to crypt ratio in the jejunum and a 12% increase of this ratio (*p =* 0.03) in the ileum compared with those fed diets without carbadox. The area of Peyer’s patches in the ileum was not affected by dietary treatments, suggesting Zn concentration and carbadox may not directly influence the development of Peyer’s patches at 7 days post-weaning. 

### 2.3. Effects of Dietary Zn and Carbadox on Ileal Cytokines

The ileal concentration of granulocyte-macrophage colony-stimulating factor (GM-CSF), IL-2, and tumor necrosis factor α (TNFα) were below the limit of detection of the cytokine panel, and thus, only the concentrations of 10 cytokines are reported (Table 2). For pro-inflammatory cytokines, pharmacological Zn supplementation and carbadox treatments reduced the concentration of IL-1β compared with basal Zn supplementation (*p =* 0.03) and non-carbadox treatments (*p <* 0.01). The concentration of IL-18 in pharmacological Zn supplementation was reduced numerically by 11% (*p =* 0.09) compared with basal Zn. Furthermore, Zn concentration and carbadox resulted in an interaction effect (*p =* 0.01) on the concentration of IL-6. The addition of carbadox reduced the concentration of IL-6 in pigs fed a pharmacological dose of Zn (*p <* 0.05), but not in pigs fed a basal dose of Zn. 

For anti-inflammatory cytokines, carbadox treatments tended to increase the concentration of IL-10 (*p =* 0.09) compared with non-carbadox treatments. Another interaction between Zn concentration and carbadox (*p =* 0.03) was observed in the concentration of IL-1RA. Adding carbadox reduced the concentration of IL-1RA (*p <* 0.05) in pigs fed a pharmacological dose of Zn, but the response was not observed in pigs fed basal dose of Zn. Finally, the ratio of anti- to pro-inflammatory cytokines showed that pharmacological Zn supplementation increased the IL-1RA:IL-1 ratio by 42% (*p =* 0.04) and carbadox treatments tended to increase IL-10:IL-12 (*p =* 0.07) compared with a basal dose of Zn and non-carbadox treatments.

### 2.4. Effects of Dietary Zn and Carbadox on Free Amino Acid (AA), Bile Acid (BA), and Fatty Acid (FA) in Ileal Digesta

Free AA, BA, and FA in ileal digesta were not affected by pharmacological Zn supplementation or carbadox (Table 3). However, the carbadox treatments tended to increase the concentrations of essential AA (i.e., Leu/Ile, Thr, and Trp) and palmitic acid in ileal digesta (*p =* 0.09, 0.07, 0.08, and 0.09, respectively) compared with non-carbadox treatments. Because AA, FA, and BA participate in a variety of physiological and metabolic functions, the correlation analysis can be useful for interpreting whether the changes of those metabolites may be related to animal growth performance responses or not. The correlations between metabolites of ileal digesta and ADG from 0–7 days post weaning were examined, but no significant correlations between ADG and BA and FA were observed. However, essential AA including Arg, His, Leu/Ile, Met, Thr, Trp, and Val in the ileal digesta were positively correlated with ADG (r = 0.38, 0.36, 0.40, 0.37, 0.44, 0.46, and 0.41, respectively, *p <* 0.05; Table 3).

### 2.5. Effects of Dietary Zn and Carbadox on Free AA, BA, and FA in Feces

Carbadox treatments selectively increased the concentration of some essential AA, including His (*p =* 0.03) and Lys (*p =* 0.03), as well as non-essential AA, including Ala (*p =* 0.01), Glu (*p <* 0.01), Ser (*p =* 0.04) in feces, while pharmacological Zn supplementation had no effects on fecal AA concentrations (Table 4). Neither pharmacological Zn supplementation nor the carbadox treatments affected the concentration of BA in feces. Similarly, pharmacological Zn supplementation and the carbadox treatments did not alter the fecal concentration of short-chain FA. However, feces from pigs fed diets containing pharmacological Zn concentrations had a greater concentration of C18:3 (*p =* 0.01) than feces from pigs fed the basal dose of Zn. Furthermore, the carbadox treatments increased fecal C14:1 (*p <* 0.01), C16:1 (*p =* 0.02), and C18:3 (*p =* 0.04) compared with the non-carbadox treatments. The correlation analysis between fecal metabolites and ADG from 0–7 days post weaning showed that Ala, Asp, Glu, Gly, C14:1 C18:1, C18:2 and C18:3 were positively correlated with ADG (r = 0.40, 0.32, 0.36, 0.34, 0.50, 0.18, 0.32 and 0.34, respectively, *p <* 0.05; Table 4). 

## 3. Discussion

**Figure 3:**
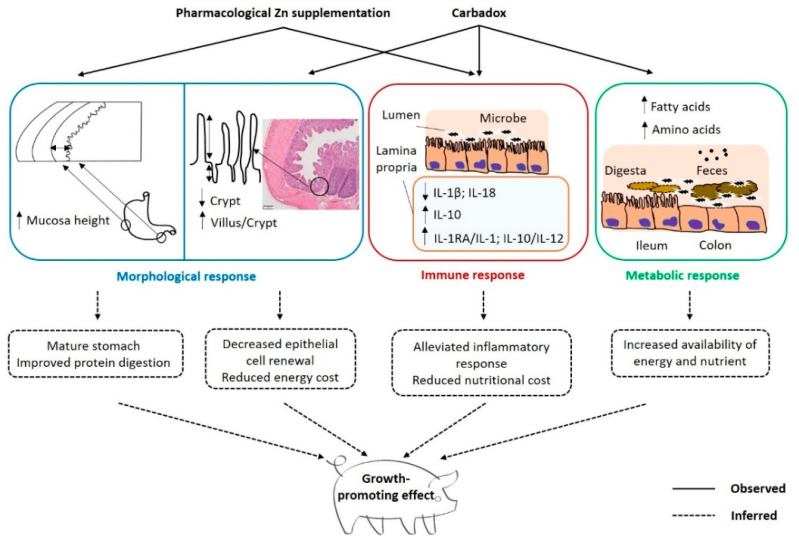
Summary the morphological, immune and metabolic responses of pharmacological Zn supplementation and carbadox to post-weaning pigs.

Post-weaning diarrhea is a common health issue associated with growth depression in nursery pigs, and was usually controlled by the addition of a pharmacological dose of Zn in diets, mainly in the form of ZnO [8,9,19]. The North Central Regional-42 Committee on Swine Nutrition has reported that adding Zn up to 3000 mg/kg in swine diets improved post-weaning growth performance in a dose-dependent manner, especially for pigs older than 28 days [12]. Additional growth-promoting benefits have also been observed when carbadox was added in combination with pharmacological Zn supplementation in diets for nursery pigs [12,20]. The results of the present study reconfirmed these growth performance responses when supplementing diets with ZnO (2500 mg/kg) and carbadox independently and in combination for nursery pigs, and no severe PWD and obvious illness were observed during the trial. More importantly, the influences of Zn and carbadox on GI morphology, intestinal immune status, and metabolites of digesta and feces were examined to identify their associations with growth performance, and summarized in Figure 3.

Gastric morphology is commonly evaluated by histological examination of corpus and antral mucosa, two main regions with distinctive cell types and functions. The corpus mucosa contains oxyntic glands secreting mucus, gastric acid, ghrelin, and enzymes, while the antral mucosa glands secrete mucus and gastrin [21,22]. In the present study, Zn effects on the growth of gastric mucosa were observed, but the exact mechanisms behind this observation required further investigation. A similar effect of Zn on intestinal mucosal growth has been reported. Potential mechanisms induced by pharmacological Zn supplementation include increased insulin-like growth factor-1 in blood [15,23,24] and increased ghrelin secretion in pigs post-weaning [15], which both can stimulate intestinal mucosa growth [15,23,24]. In contrast to the observation on gastric mucosa, intestinal villus height was not affected by pharmacological Zn supplementation in the present study. Increased villus height effects were reported in some studies when diets containing pharmacological doses of Zn were fed to weaned pigs [16,25], but not in other studies [26,27]. 

In contrast to the effects of pharmacological Zn supplementation on gastric and intestinal morphology, carbadox treatments did not affect stomach mucosa, but decreased crypt depth and increased villus:crypt ratio in the small intestine. This observation is in agreement with the results from feeding AGPs to broilers [28]. As an antimicrobial, carbadox mainly targets the gut microbiome in the host. Decreases in the richness of gut microbial taxa as well as individual species, such as *E. coli,* have been observed in pigs fed diets containing carbadox [29]. It has been reported that the gut microbiota can change intestinal morphology. For example, enterotoxigenic *E. coli* that causes PWD can significantly reduce villus:crypt ratio of weaned pigs [30]. Therefore, the carbadox treatment used in the current study may have resulted in decreased crypt depth and increased intestinal villus:crypt ratio through modifications of gut bacterial communities. The intestinal stem cells reside in the crypt and produce transient amplifying cells that can differentiate into different cell lineages [31]. Therefore, shallow crypts with unchanged villi height, indicated by increased villus:crypt ratio in the jejunum and ileum, may indicate stable, low cell turnover that results in lower energy requirements for intestinal cell maintenance and regeneration, leading to sparing energy and amino acids for overall growth. 

Niewold (2007, 2014) suggested that the growth-promoting effects of AGPs are likely due to the inhibition of the intestinal inflammatory responses [13,32]. The status of inflammatory responses can be evaluated effectively by the individual cytokines and the ratios among cytokines. For example, the IL-10:IL-12 ratio indicates the balance between anti-inflammatory and pro-inflammatory signals [33]. Weaning is commonly associated with intestinal inflammation in piglets, as indicated by the upregulation of mRNA expression of IL-1β, IL-6, and TNF-α [34]. We analyzed the concentration of anti- and pro-inflammatory cytokines in the intestine to directly address the intestinal response that cannot be discerned from serum measurements [35]. In the current study, both pharmacological Zn supplementation and carbadox treatments reduced the concentration of IL-1β, which could be beneficial for animal growth because the nutritional cost of inflammation is responsible for the animal’s growth potential [36]. Pharmacological Zn supplementation increased IL-1RA:IL-1 ratio, suggesting an ability of Zn to alleviate pro-inflammatory responses because of a negative correlation between IL-1RA:IL-1 ratio and inflammatory bowel disease [37,38]. Carbadox tended to increase the IL-10:IL-12 ratio, which suggests the ability of carbadox to reduce Th1 cytokines (pro-inflammatory cytokines) [33]. Overall, these results suggest that pharmacological Zn supplementation and carbadox alleviate host intestinal immune responses by reducing pro-inflammatory cytokines and stabilizing the anti- to pro-inflammatory cytokines ratio, which may contribute to less metabolic cost for immune responses and result in more energy and nutrients being available for growth. 

Amino acids, FA, and BA are the major functional metabolites in the GI tract because they serve as the sources of energy and nutrients for growth and also regulate inflammation and signaling. Our analysis focused on metabolites instead of microbiome composition in the GI tract and addressed functional changes induced by pharmacological Zn and carbadox. The correlation analysis in the current study confirmed the relevance of their concentration, especially AA concentrations, in ileal digesta and feces to growth performance. Interestingly, essential AA in ileal digesta, including Leu/Ile, Val, Trp, Thr, Arg, and His, had greater correlations with ADG than other AA. In contrast, in feces, non-essential AA, including Ala, Gly, Glu, and Asp, were better correlated with ADG than essential AA. The correlation of essential AA in ileal digesta with growth is not unexpected based on their nutritional value, but the correlation of nonessential AA in feces with growth is not a well-established fact and requires further investigation. Nevertheless, the causes of correlation between fecal AA and growth should be different from correlations between ileal AA and growth responses in considerations of the distinct physiological role and composition of microbiota.

Analyzing the influence of Zn concentration and carbadox on these metabolites in ileal digesta and feces revealed two prominent features: (1) carbadox treatments caused far more metabolic changes than pharmacological Zn supplementation, and (2) many metabolic changes from the carbadox treatments were only observed in feces, but not in ileal digesta. Because the large intestine is the physiological connection between the ileal digesta and feces, these features clearly suggest that anti-microbial activity of carbadox in the large intestine may be responsible for the changes in fecal metabolites. 

Microbial protein constitutes a large proportion of fecal protein in piglets [39]. The increases of certain free AA in fecal samples from pigs fed carbadox in the current study should be not interpreted as a reduction in protein digestibility. Instead, it should be considered a consequence of increased microbial fermentation of proteins or increased microbial synthesis of AA in the large intestine, considering that the protein concentrations were similar between experimental diets within each phase. This phenomenon has been observed in mouse studies, in which fecal Val, Leu, Ile, and Phe levels were increased by feeding diets containing ciprofloxacin, while Arg and Lys were increased by feeding vancomycin-imipenem compared with no antibiotic treatment [40]. The exact mechanisms for these selective effects of carbadox on fecal AA remain to be determined. However, the microbial synthesis of essential AA in pigs, including Lys, Phe, Leu, Ile, and Val, has been reported previously [41], and the increased expression of the microbial genes encoding aromatic AA has also been observed in the pigs fed antibiotic cocktails [42]. It is important to mention that the colonocytes may have potential to absorb a small amount of AA due to the presence of AA transporters [43]. Therefore, all of these activities can increase nutrient and energy utilization in nursery pigs and provide the substrates for producing other functional metabolites, including AA, short-chain FAs, organic acids, phenolic and indole compounds [44].

Short-chain FAs and secondary BAs are two groups of microbial metabolites commonly examined in antibiotic studies. Selective effects of antibiotic treatments on short-chain FAs and secondary BAs have been observed in previous studies. For example, Looft et al. found that feeding carbadox increased the relative abundance of *Prevotella*, a genus of bacteria involved in producing short-chain FAs, in the microbiome of weaned pigs [29], while Trudeau et al. reported that feeding with tylosin did not affect short-chain FAs, but selectively increased hyodeoxycholic acid, a secondary BA in feces [45]. In the current study, neither short-chain FAs nor BAs were affected by pharmacological Zn supplementation and carbadox treatments. The inverse correlation between ADG and ileal lithocholic acid (LCA), a secondary BA, was observed. LCA exposure has been shown to impede Th1 cell activation *in vitro* [46]. However, this correlation might not be able to explain the observed changes in cytokines because LCA is a very minor secondary BA in ileal digesta in comparison to other BAs. Interestingly, selective medium-chain and long-chain FAs were affected by the treatments. Pharmacological Zn supplementation increased pentadecanoic acid (C15:0) and α-linolenic acid (C18:3), while carbadox addition increased caproic acid (C6:0), myristoleic acid (C14:1), palmitoleic acid (C16:1), and α-linolenic acid (C18:3) in feces. These metabolic changes can likely be attributed to microbial metabolism, but the exact mechanisms remain to be determined. 

Overall, the independent and additive effects of pharmacological Zn supplementation and carbadox on growth performance of weaned pigs provided proof of concept that their mode of action on growth promotion occur likely through eliciting different responses. Our findings demonstrated that pharmacological Zn and carbadox effects on morphology, immune, and metabolic status of the GI tract were different. Pharmacological Zn supplementation affected gastric mucosa while carbadox addition affected intestinal mucosa and modified the presence of targeted metabolites, and both influenced intestinal immune status (Figure 3). These results further confirmed the concept that beneficial effects of growth-promoting agents are multifactorial, including GI morphology, immune response, and microbial metabolism, suggesting that prospective alternatives for AGPs and a pharmacological dose of Zn should manifest similar effects on these parameters.

## 4. Materials and Methods

All chemicals used were purchased from Sigma-Aldrich unless otherwise stated. The feeding experimental procedures were conducted in the Cargill Animal Nutrition Innovation Campus (Elk River, MN, USA), approved and supervised by Cargill Animal Nutrition Committee on Animal Use for Research and Scientific Purposes in accordance with Directive 2010/63/EU. All other analysis and procedures were performed at the University of Minnesota (St. Paul, MN, USA) and performed in accordance with the University’s regulation and guidelines.

### 4.1. Animals and Experimental Design

A total of 192 crossbred barrows and gilts (BW = 5.9 ± 1.1 kg) were weaned at 21 days of age and fed four experimental diets during a 43-day feeding period, using a three-phase feeding program (Phase 1 = day 0 to day 7, Phase 2 = day 7 to day 21, and Phase 3 = day 21 to day 43, post weaning). Pigs were housed individually in 4 identical nursery rooms and stratified by sex and BW into 8 blocks. The experimental design consisted of a 2 × 2 factorial arrangement of treatments in a randomized complete block design. The first factor was dietary concentration of Zn to meet the requirement (basal Zn; 150 mg/kg diet) and pharmacological dose (2500 mg Zn/kg diet). The second factor was carbadox (Mecadox; Phibro Animal Health, Fairfield, NJ, USA) added to the diets at a concentration of 0 or 55 mg/kg diet. The pharmacological dose of Zn was provided from ZnO and was removed in the third phase of the feeding program. All diets were formulated to meet or exceed the nutritional requirements of weaned pigs suggested by Cargill Nutrition System (Table S2). Pigs were provided ad libitum access to their assigned experimental diets and water during the entire study. 

### 4.2. Data and Sample Collection

Experimental diet samples were collected and analyzed by Cargill Animal Nutrition with Near Infrared Spectroscopy at the Cargill Animal Nutrition Innovation Center (Elk River, MN) for ash, crude protein, ether extract, neutral detergent fiber, and moisture. Focal pigs (*n* = 10/treatment) were selected by statisticians with consideration of BW, sex, and room. In order to characterize early physiological changes, focal pigs were euthanized by CO_2_ on day 7 post-weaning (28 d of age) for collecting tissue samples. About 20 mL of gastric contents was collected and stored at −20 °C for gastric acidity determination. Samples of gastric antrum and corpus, jejunum (1 m distal to the pyloric sphincter), and ileum (15 cm proximal to the ileocecal valve) were collected and fixed in 4% buffered formaldehyde (Fisher Scientific, Hampton, VA, USA) for histological evaluation. About 2 g of ileal digesta from focal pigs and feces from rectum (*n* = 44 pigs/treatment) were also collected, snap frozen, and stored at −80 °C for analysis of targeted metabolites. 

### 4.3. Gastric Acid Titration

Gastric acid was analyzed using the protocol as described by Waghray et al. [47]. Briefly, thawed gastric contents were centrifuged at 3000× *g* for 15 min at 4 °C, and the supernatant was recovered for acid titration with an automatic titrator (TitroLine easy, Schott Instruments GmbH, Germany). The supernatants were diluted 10 × with 0.9% saline solution and titrated with 0.01 N sodium hydroxide (NaOH) solution using the titrator. Each sample was analyzed in triplicate and the volume of NaOH used to titrate to a final pH of 7.4 was recorded. Gastric acidity was expressed as mEq H+/mL and calculated by the formula: ((volume of NaOH of sample − volume of NaOH of blank) × normality of the titrating solution)/sample volume) × sample dilution factor. 

### 4.4. Histological Analysis

Tissue samples were fixed overnight in 4% buffered formaldehyde and trimmed before processing in a STP 120 Spin Tissue Processor (Thermo Fisher Scientific, Waltham, MA, USA). Tissues were then embedded in paraffin, and tissue blocks were sectioned at 5 µm thickness and mounted on charged slides. Deparaffinized slides were stained with hematoxylin and eosin (H&E, Newcomer Supply, Middleton, WI, USA). Stomach antrum and corpus sections were also stained with periodic acid–Schiff with Alcian blue (PAS-AB, Newcomer Supply, Middleton, WI, USA) following the manufacturer’s instructions after rehydration. Histological analysis was performed as previously described [48]. Briefly, the total mucosal height of corpus and antrum were measured from the tip of the glands to the muscularis mucosa. Intestinal villus height was defined as the length of a line drawn at the center of the villus from the crypt neck to the tip of the villus. Villus width was measured as the distance between the epithelial cells at the middle point between the tip and the base of the villus. Crypt depth was measured as the distance at the center of the crypt from the villus neck toward the muscularis mucosa up to the point where epithelial cells were observed. Peyer’s patches were defined as round to oval follicular nodes. Well-oriented mucosal glands and villi were measured in 10 randomly chosen fields per slide at 100 × magnification and the area of each Peyer’s patch was measured at 40 × magnification under light microscopy (Olympus BX53, Center Valley, NJ, USA), using the CellSense image software for measuring (Olympus, Center Valley, NJ, USA). Data presented are the means of the 10 fields for each pig in each treatment.

### 4.5. Tissue Cytokine Analysis

Tissue cytokine analysis was performed as previously described by Ferrandis Vila et al. [49]. Briefly, tissue protein was extracted after homogenization of ileal samples in lysis buffer containing deoxycholic acid (12.7 mM), Igepal CA-630 (1%), Tris-HCl (50 mM), NaCl (150 mM), and protease inhibitor cocktail (1X, Halt protease inhibitor Cocktail, Thermo Fisher Scientific, Rockford, IL, USA) adjusted to pH 7.4. Homogenized samples were centrifuged at 12,000× *g* for 15 min at 4 °C and total protein was quantified using a NanoDrop 2000 (Thermo Scientific, Wilmington, DE, USA) and stored at −80 °C until cytokine analyses. A Multiplex Map Kit (Porcine cytokine/chemokine Magnetic Bead Panel, Merck Millipore, Darmstadt, Germany) was used to quantify 13 porcine cytokines, including granulocyte-macrophage colony-stimulating factor (GM-CSF), interferon γ (IFNγ), interleukin (IL)-1α, IL-1β, IL-1 receptor antagonist (IL-1RA), IL-2, IL-4, IL-6, IL-8, IL-10, IL-12, IL-18, and tumor necrosis factor α (TNFα). The concentration of each cytokine was expressed per mg of total tissue protein.

### 4.6. Quantitative Analysis of Free Amino Acids, Fatty Acids, and Bile Acids in Ileal Digesta and Feces

Sample preparation. Ileal fluid was obtained by centrifuging the digesta, and the fluid was further diluted 5 times with 50% aqueous acetonitrile (ACN) followed by centrifugation at 18,000× *g* for 10 min to obtain the supernatants. Fecal samples were soaked in 50 volumes (*v*/*w*) of 50% aqueous ACN overnight at 4 °C, extracted by vortexing and sonication for 10 min, and centrifuged at 18,000× *g* for 10 min to remove the insoluble fraction [45].

Chemical derivatization. For free AA analysis, samples were derivatized by dansyl chloride (DC) [50]. Briefly, 5 μL of sample or standard was mixed with 5 μL of 100 μM *p*-chlorophenylalanine (internal standard), 50 μL of 10 mM sodium carbonate, and 100 μL of DC solution (3 mg/mL in acetone). The mixture was incubated at 60 °C for 15 min, centrifuged at 18,000× *g* for 10 min, and the supernatant was transferred into a vial for further analysis. For FA analysis, samples were derivatized by 2-hydrazinequinoline (HQ) [51]. Briefly, 2 μL of sample was added into a 100 μL of freshly prepared ACN solution containing 1 mM 2,2′-dipyridyl disulfide, 1 mM triphenulphosphine, and 1 mM HQ. The reaction mixture was incubated at 60°C for 30 min, chilled on ice, and then mixed with 100 μL of ice-cold deionized water. After centrifugation at 18,000× *g* for 10 min, the supernatant was transferred into a vial for analysis. The DC and HQ derivatives in the reaction mixture were analyzed using Liquid Chromatography–Mass Spectrometry (LC–MS). 

LC–MS analysis. The analysis was performed as described previously [50]. A 5 μL aliquot prepared from the sample of ileal fluid or fecal extract was injected into an Acquity Ultra-Performance Liquid Chromatography system (Waters, Milford, MA) and separated in a BEH C18 column. For BA analysis, 10 mM NH4OAc (pH = 9) (A), 95% ACN and 5% water with 10 mM NH4OAc (pH = 9) (B) were used as mobile phase. For AA analysis, 0.1% formic acid (A) and ACN containing 0.1% formic acid (B) were used as mobile phase. Fatty acid analysis was conducted using 0.05% (*v*/*v*) aqueous acetic acid containing 5 mM ammonium acetate (A), 95% (*v*/*v*) aqueous ACN containing 0.05% acetic acid, and 5 mM ammonium acetate (B) as the mobile phase. The eluent from LC was introduced into a mass spectrometer (Q-TOF-MS, Waters) for accurate mass measurement and ion counting. For electrospray ionization, capillary voltage and cone voltage were maintained at 3 kV and 30 V for positive-mode detection or at 3 kV and 35 V for negative-mode detection, respectively. Source temperature and desolvation temperature were set at 120 °C and 350 °C, respectively. Nitrogen was used as both cone gas (50 L/h) and desolvation gas (600 L/h), with argon used as the collision gas. Sodium formate solution (mass-to-charge (m/z) ratio: 50–1000) was used to calibrate the mass spectrometer and was monitored by the intermittent injection of the lock mass leucine enkephalin ([M + H]+ = 556.2771 m/z) in real time. Mass chromatograms and mass spectral data were acquired and processed by MassLynxTM software in centroided format. Additional structural information was obtained by tandem MS (MS/MS) fragmentation with collision energies ranging from 15 to 40 eV. 

Data analysis. The concentration of individual metabolites was determined by calculating the ratio between the peak area of compound and the peak area of internal standard, and a standard curve was fitted using QuanLynx software (Waters, Milford, MA, USA).

### 4.7. Statistical Analysis

To calculate sample size, growth performance was considered the primary outcome and a power calculation was performed in SAS (v9.3; SAS Inst. Inc., Cary, NC, USA) using an effect size of 29.7 g for ADFI and 26.7 g for ADG, α level of 0.05, and a power of 80%. The calculation indicated a minimal number of pigs per group of 38. Forty-eight pigs were used per treatment to account for possible losses along the experiment. The normality test was evaluated using the procedure (PROC) Univariate procedure of SAS, and data were analyzed as a randomized complete block design using the PROC Mixed procedure. Because pigs were housed individually, pigs were considered as experimental unit for all measurements and were used as a subject for repeated measurements for data analysis of growth performance responses. Fixed effects included dietary concentration of Zn, addition of carbadox, time, and two- and three-way interactions for growth performance responses. Block was considered as a random effect. In contrast to growth performance, the fixed effect of time was removed from the statistical models for other responses, including gastric acidity, histological analysis, tissue cytokines analysis, and quantification of AA, FA, and BA analysis. Data for growth performance were analyzed using repeated measurements in the model. All mean values were reported as least squares means. Multiple comparisons among treatments were performed using *p*-values for differences (PDIFF) and adjusted by Tukey for multiple comparisons of means. Additionally, the PROC correlation (CORR) procedure of SAS was applied to identify correlations between metabolites and ADG. Significant differences were declared at *p* < 0.05 and statistical trends at *p* < 0.10. 

### 4.8. Ethical Approval

The project has been approved by the Cargill Animal Nutrition Animal Care and Use Committee—Elk River (Project identification code: 2577-1829N).

## Figures and Tables

**Table 1:** Gastric acid production and gastrointestinal (GI) morphology of post-weaning pigs fed diets containing a pharmacological dose of Zn and carbadox.

Zn	150		2500		SE ^1^	*p*-values		
Carbadox	No	Yes	No	Yes		Zn	AB ^2^	Zn × AB ^3^
**Stomach**								
Mucosal height of corpus, µm	671.5	606.7	736.3	723.2	27.52	<0.01	0.13	0.33
Mucosal height of antrum, µm	496.8	489.6	561.8	534.5	15.11	<0.01	0.25	0.50
Gastric acid, mEq/mL	0.065	0.054	0.058	0.052	0.006	0.49	0.19	0.71
**Jejunum**								
Villus width, μm	93.4	85.0	90.7	93.0	5.35	0.56	0.50	0.25
Villus height, μm	535.6	500.8	551.6	572.3	23.74	0.07	0.77	0.25
Crypt depth, μm	143.1	113.9	139.3	137.7	5.63	0.08	0.01	0.02
Villus:crypt ratio	3.74 ^a^	4.40 ^b^	3.99 ^ab^	4.17 ^ab^	0.09	0.94	<0.01	0.06
**Ileum**								
Villus width, μm	71.1	69.3	68.2	73.5	2.7	0.81	0.51	0.19
Villus height, μm	280.4	286.9	287.8	308.6	16.3	0.38	0.41	0.67
Crypt depth, μm	111.0 ^b^	90.1 ^a^	98.2 ^ab^	99.4 ^ab^	5.4	0.75	0.07	0.05
Villus:Crypt ratio	2.64	3.22	2.93	3.10	0.17	0.61	0.03	0.22
Peyer’s patch, μm ^2^	163,673	169,555	171,699	163341	9816	0.93	0.90	0.47

^a,b,c^ Values in the same row with different superscript differ (*p <* 0.05); ^1^ SE = pooled standard error of means, *n* = 10 pigs/treatment; ^2^ AB = Carbadox; ^3^ Interaction effect between Zn concentration and Carbadox.

**Table 2:** Concentration of ileal cytokines of pigs fed diets containing a pharmacological dose of Zn and Carbadox ^1^.

Zn	150		2500		SE ^2^	*p*-values		
Carbadox	No	Yes	No	Yes		Zn	AB ^3^	Zn × AB ^4^
IFNγ	96.70	85.24	91.06	84.39	10.04	0.75	0.38	0.81
IL-1α	1.19	1.18	1.16	0.90	0.19	0.38	0.43	0.46
IL-1β	85.27	44.33	51.78	34.48	9.75	0.03	<0.01	0.23
IL-1RA	13.21 ^ab^	12.89 ^ab^	18.1 ^b^	11.24 ^a^	1.48	0.28	0.02	0.03
IL-1RA:IL-1	0.20	0.32	0.39	0.35	0.05	0.04	0.41	0.13
IL-4	19.74	15.07	15.39	8.48	7.05	0.46	0.43	0.88
IL-6	1.27 ^a^	2.29 ^ab^	3.32 ^b^	1.34 ^a^	0.68	0.28	0.33	0.01
IL-8	270.47	234.71	224.13	170.08	49.36	0.12	0.20	0.80
IL-10	0.36	0.54	0.31	0.58	0.12	0.98	0.09	0.69
IL-12	2.67	2.36	2.89	2.12	0.56	0.98	0.34	0.68
IL-10:IL-12	0.14	0.26	0.15	0.23	0.05	0.83	0.07	0.67
IL-18	931.05	889.50	856.91	765.01	57.45	0.09	0.25	0.66

^a,b,c^ Values in the same row with different superscript differ (*p <* 0.05); ^1^ Units are pg/mg total protein for all cytokines except for IL-4, which is fg/mg total protein; ^2^ SE = pooled standard error of means, *n* = 10 pigs/treatment; ^3^ AB = Carbadox; ^4^ Interaction effect between Zn concentration and Carbadox.

**Table 3:** Effect of Zn concentration and carbadox on amino acid (AA), fatty acid (FA), and bile acid (BA) in ileal digesta.

Zn	150		2500		SE ^1^	*p-*values			ADG (r Value) ^4^
Carbadox	No	Yes	No	Yes		Zn	AB ^2^	Zn × AB ^3^	
**Amino acids, mg/g**									
Alanine	0.257	0.252	0.221	0.270	0.030	0.78	0.51	0.41	0.33
Arginine	0.321	0.282	0.290	0.595	0.108	0.20	0.23	0.12	0.38
Asparagine	0.035	0.081	0.070	0.080	0.027	0.53	0.28	0.49	0.32
Aspartic acid	0.131	0.169	0.149	0.220	0.034	0.31	0.11	0.64	-
Glutamic acid	1.011	1.087	0.965	1.112	0.174	0.95	0.53	0.84	-
Glutamine	0.123	0.142	0.113	0.184	0.035	0.64	0.20	0.45	0.33
Glycine	0.211	0.255	0.191	0.204	0.035	0.31	0.42	0.66	-
Histidine	0.070	0.073	0.064	0.093	0.015	0.63	0.29	0.38	0.36
Leucine/Isoleucine	0.195	0.216	0.152	0.265	0.039	0.94	0.09	0.25	0.40
Lysine	0.729	0.612	0.687	0.818	0.132	0.54	0.96	0.35	-
Methionine	0.021	0.028	0.016	0.033	0.009	0.94	0.19	0.57	0.37
Phenylalanine	0.259	0.257	0.192	0.289	0.038	0.64	0.22	0.20	-
Proline	0.201	0.163	0.157	0.199	0.024	0.87	0.92	0.11	0.30
Serine	0.086	0.128	0.113	0.163	0.036	0.39	0.21	0.91	0.38
Threonine	0.073	0.103	0.075	0.133	0.023	0.45	0.07	0.55	0.44
Tryptophan	0.043	0.063	0.047	0.095	0.019	0.35	0.08	0.46	0.46
Tyrosine	0.304	0.294	0.236	0.391	0.069	0.84	0.30	0.24	0.35
Valine	0.174	0.192	0.141	0.241	0.035	0.83	0.11	0.26	0.41
**Fatty acids, mg/g**									
Acetic acid	0.376	0.159	0.148	0.221	0.069	0.20	0.27	0.03	-
Propionic acid	0.115	0.008	0.002	0.001	0.054	0.35	0.32	0.34	-
Butyric acid	0.638	0.018	0.010	0.013	0.311	0.32	0.33	0.33	-
C6:0	0.130	0.093	0.098	0.085	0.020	0.32	0.22	0.53	-
C8:0	0.008	0.007	0.009	0.008	0.001	0.35	0.45	0.71	-
C10:0	0.002	0.002	0.009	0.010	0.002	0.24	0.73	0.73	-
C12:0	0.060	0.061	0.049	0.029	0.027	0.42	0.73	0.70	-
C14:0	0.035	0.061	0.024	0.035	0.015	0.25	0.23	0.65	-
C14:1	0.009	0.005	0.004	0.006	0.003	0.57	0.78	0.30	-
C15:0	0.009	0.016	0.008	0.009	0.003	0.24	0.25	0.41	-
C16:0	1.944	2.371	2.214	2.419	0.186	0.38	0.09	0.55	-
C16:1	1.044	0.895	0.498	0.825	0.309	0.33	0.78	0.45	-
C17:0	0.019	0.042	0.024	0.023	0.011	0.52	0.34	0.30	-
C17:1	0.089	0.075	0.035	0.065	0.025	0.21	0.75	0.39	-
C18:0	4.162	3.314	3.999	3.425	0.423	0.95	0.10	0.75	-
C18:1	7.775	8.369	7.792	9.257	2.250	0.83	0.62	0.84	-
C18:2	6.029	5.992	5.467	6.364	1.674	0.95	0.79	0.77	-
C18:3	0.830	0.890	0.770	0.870	0.310	0.90	0.79	0.96	-
**Bile acids,** **μg/g**									
LCA	0.092	1.001	0.166	0.067	0.397	0.27	0.30	0.20	−0.32
CDCA	7.67	16.47	14.50	2.85	6.980	0.63	0.84	0.15	-
CA	0.144	4.615	0.404	0.202	1.513	0.17	0.15	0.12	-
GDCA	0.047	3.809	0.088	0.309	1.835	0.35	0.29	0.34	-
TCDCA	4.13	11.05	5.72	2.78	4.278	0.44	0.64	0.26	-
DCA	1.33	1.57	2.45	1.21	0.804	0.64	0.54	0.36	-
GCA	0.242	10.031	0.584	0.514	3.038	0.14	0.12	0.11	-
GCDCA	14.31	26.85	13.87	17.42	12.86	0.70	0.54	0.73	-
TDCA	1.991	1.202	1.830	2.496	0.954	0.56	0.95	0.45	-
TCA	1.499	3.347	0.500	0.210	1.597	0.20	0.63	0.50	-
HDCA	25.24	6.33	38.67	22.72	14.84	0.25	0.17	0.91	-

^1^ SE = pooled standard error of means, *n* = 10 pigs/treatment; ^2^ AB = Carbadox; ^3^ Interaction effect between Zn concentration and Carbadox; ^4^ Pearson correlation coefficient of metabolite to ADG day 0 – 7 (only correlations with *p <* 0.05 are presented).

**Table 4:** Effect of Zn concentration and carbadox on AA, FA, and BA in feces.

Zn	150		2500		SE ^1^	*p-*values			ADG (r Value) ^4^	
Carbadox	No	Yes	No	Yes		Zn	AB ^2^	Zn × AB ^3^		
**Amino acids, mg/g**										
Alanine	0.056	0.081	0.063	0.069	0.007	0.77	0.01	0.13	0.40	
Arginine	0.006	0.010	0.011	0.008	0.002	0.60	0.69	0.10	-	
Asparagine	0.001	0.002	0.001	0.001	0.001	0.60	0.38	0.32	-	
Aspartic acid	0.050	0.054	0.052	0.059	0.006	0.55	0.31	0.79	0.32	
Glutamic acid	0.357	0.493	0.387	0.490	0.046	0.73	<0.01	0.68	0.36	
Glutamine	0.007	0.009	0.007	0.009	0.002	0.94	0.20	0.90	-	
Glycine	0.048	0.076	0.049	0.055	0.009	0.29	0.07	0.22	0.34	
Histidine	0.011	0.047	0.010	0.013	0.009	0.05	0.03	0.07	-	
Leucine/Isoleucine	0.148	0.179	0.148	0.172	0.017	0.83	0.10	0.85	-	
Lysine	0.315	0.426	0.351	0.386	0.033	0.94	0.03	0.25	-	
Methionine	0.026	0.030	0.025	0.029	0.003	0.85	0.21	0.94	-	
Phenylalanine	0.128	0.156	0.121	0.132	0.020	0.42	0.32	0.68	-	
Proline	0.071	0.073	0.083	0.074	0.009	0.47	0.71	0.58	-	
Serine	0.050	0.060	0.052	0.059	0.005	0.88	0.04	0.72	-	
Threonine	0.028	0.034	0.029	0.035	0.003	0.85	0.07	0.93	-	
Tryptophan	0.022	0.018	0.014	0.016	0.005	0.28	0.77	0.55	-	
Tyrosine	0.175	0.178	0.158	0.180	0.024	0.76	0.61	0.70	-	
Valine	0.089	0.109	0.090	0.104	0.009	0.83	0.06	0.77	-	
**Fatty acids, mg/g**										
Acetic acid	3.549	3.310	3.593	3.721	0.270	0.39	0.85	0.49	0.31	
Propionic acid	2.020	2.028	2.286	2.399	0.250	0.14	0.78	0.81	-	
Butyric acid	4.482	3.856	4.225	4.844	0.474	0.44	0.99	0.19	-	
C6:0	0.753	0.828	0.631	1.313	0.163	0.22	0.01	0.04	-	
C8:0	0.016	0.016	0.015	0.017	0.001	0.91	0.45	0.42	-	
C10:0	0.009	0.011	0.011	0.013	0.001	0.11	0.14	0.76	-	
C12:0	0.214	0.182	0.197	0.164	0.023	0.43	0.15	0.97	-	
C14:0	0.350	0.351	0.443	0.442	0.049	0.06	1.00	0.98	-	
C14:1	0.020	0.031	0.023	0.035	0.005	0.30	< 0.01	0.89	0.50	
C15:0	2.149	1.967	2.931	2.724	0.326	0.02	0.55	0.97	-	
C16:0	2.931	2.592	3.317	2.975	0.205	0.06	0.10	0.99	-	
C16:1	1.705	2.107	1.536	2.065	0.216	0.60	0.02	0.75	-	
C17:0	1.430	0.838	2.000	1.591	0.341	0.05	0.14	0.79	-	
C17:1	2.465	3.213	3.370	3.805	0.876	0.35	0.46	0.84	-	
C18:0	4.603	3.862	4.658	4.034	0.347	0.74	0.05	0.86	−0.23	
C18:1	15.629	17.527	17.140	19.417	1.396	0.20	0.11	0.89	0.18	
C18:2	5.462	5.976	6.034	7.150	0.567	0.10	0.13	0.57	0.32	
C18:3	0.756	0.907	0.982	1.248	0.114	0.01	0.04	0.59	0.34	
**Bile acids, μg/g**										
LCA	185.07	182.63	160.94	233.36	25.754	0.61	0.18	0.15	-	
CDCA	8.310	11.720	12.719	10.573	3.952	0.68	0.87	0.48	-	
CA	4.070	6.159	7.064	4.808	1.049	0.44	0.94	0.04	-	
GDCA	2.327	2.720	2.962	3.199	0.398	0.16	0.42	0.84	-	
TCDCA	1.152	1.754	1.207	1.534	0.331	0.80	0.16	0.68	-	
DCA	6.933	10.936	10.364	8.194	2.903	0.91	0.75	0.29	-	
GCA	0.516	0.651	0.588	0.516	0.100	0.72	0.72	0.24	-	
GCDCA	0.781	0.972	0.965	1.014	0.138	0.39	0.36	0.59	-	
TDCA	0.308	0.424	0.288	0.719	0.196	0.48	0.16	0.42	-	
TCA	0.048	0.080	0.078	0.069	0.019	0.61	0.54	0.26	-	
HDCA	129.23	120.74	140.32	131.94	17.58	0.53	0.63	1.00	-	

^1^ SE = pooled standard error of means, *n* = 44 pigs/treatment; ^2^ AB = Carbadox; ^3^ Interaction effect between Zn concentration and Carbadox; ^4^ Pearson correlation coefficient of metabolite to ADG day 0–7 (only correlations with *p <* 0.05 are presented).

## Supplementary Materials

The following are available online at https://www.mdpi.com/2079-6382/9/8/463/s1, Table S1: Performance of post-weaning pigs fed diets containing a pharmacological dose of Zn and carbadox, Table S2: Ingredient and nutrient composition of experimental diets (as-fed basis).
